# Remodeling immune microenvironment in periodontitis using resveratrol liposomes as an antibiotic-free therapeutic strategy

**DOI:** 10.1186/s12951-021-01175-x

**Published:** 2021-12-20

**Authors:** Junyu Shi, Yi Zhang, Xiaomeng Zhang, Ruiying Chen, Jianxu Wei, Jiazhen Hou, Bing Wang, Hongchang Lai, Yongzhuo Huang

**Affiliations:** 1 Department of Implant Dentistry, Shanghai Ninth People’s Hospital, Shanghai Jiao Tong University School of MedicineCollege of Stomatology, Shanghai Jiao Tong University; National Center for Stomatology, National Clinical Research Center for Oral Diseases; Shanghai Key Laboratory of Stomatology, 639 Zhizaoju Road, Shanghai, 200011 China; 2 State Key Laboratory of Drug Research, Shanghai Institute of Materia Medica, Chinese Academy of Sciences, 501 Haike Rd, Shanghai, 201203 China; 3 Zhongshan Institute for Drug Discovery, SIMM, CAS, Zhongshan, 528437 China; 4 NMPA Key Laboratory for Quality Research and Evaluation of Pharmaceutical Excipients, Shanghai, 201203 China; 5 Taizhou University, School of Advanced Study, Institute of Natural Medicine and Health Product, Taizhou, 318000 China

**Keywords:** Resveratrol, Macrophage, Periodontitis, Liposome, Immune microenvironment, Local delivery

## Abstract

**Background:**

Periodontitis is a complicated inflammatory disease that damages the tooth-supporting tissues, with limited pharmacotherapy available. Macrophage-targeting therapy is promising for inflammatory diseases. Resveratrol (RSV), a nonflavonoid polyphenol, is known for its anti-inflammatory and immunomodulatory effects. However, its medical application is limited by its poor stability and water-solubility, as well as its low bioavailability.

**Result:**

A therapeutic resveratrol-loaded liposomal system (Lipo-RSV) was developed to treat periodontitis. The physical properties of Lipo-RSV and its ability to regulate macrophages were investigated. The results showed that Lipo-RSV had good biocompatibility and could re-educate the inflammatory macrophages from M1- to M2-like phenotype through activating p-STAT3 and downregulating p-STAT1. Besides, the Lipo-RSV could scavenge ROS and inhibit the NF-κB signal and inflammasomes, thereby reducing the pro-inflammatory cytokines IL-1β, IL-6, and TNF-α.

**Conclusion:**

These results revealed that Lipo-RSV could be a potential therapeutic system for the antibiotic-free treatment for periodontal diseases.

**Graphical Abstract:**

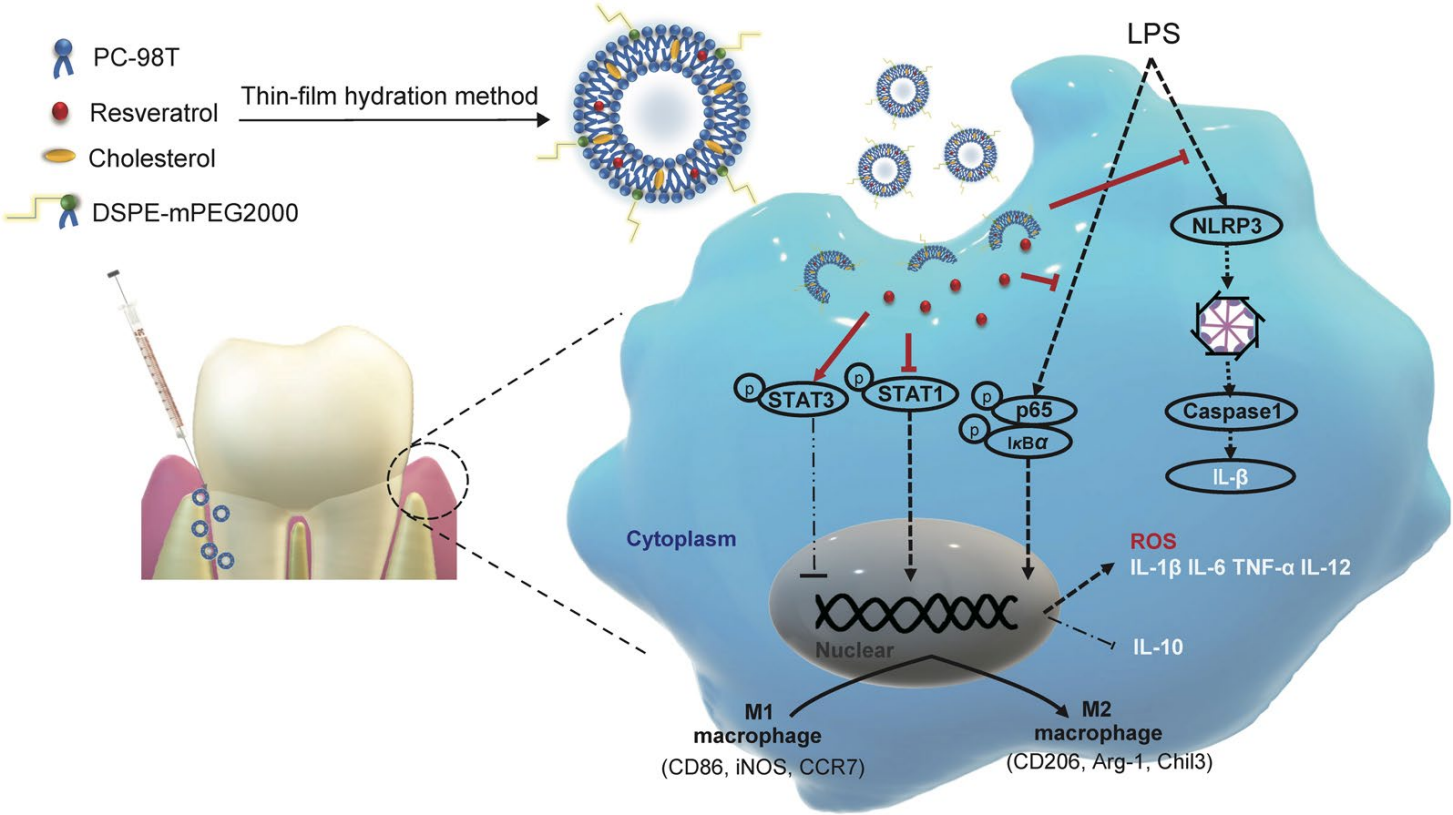

**Supplementary Information:**

The online version contains supplementary material available at 10.1186/s12951-021-01175-x.

## Introduction

As one of the most prevalent oral diseases, periodontitis can affect up to 90% of the global population, and it has been identified as a leading cause of tooth loss in adults [1]. Periodontal pockets act as bacterial niches and provide favorable conditions for microbial proliferation. Periodontitis also raises the risk of systemic diseases such as cardiovascular diseases, Alzheimer's disease, and inflammatory bowel diseases [2]. Mechanical debridement and adjunctive antibiotics remain the mainstay of treatment. However, the treatment outcomes are often compromised by the development of antibiotic resistance and drug interactions [3]. Also, the heavy use of antibiotics could further exacerbate the global crisis of antibiotic abuse [4]. Therefore, it is urgent to find new alternative treatment strategies for periodontitis [5].

Although plaque is considered to be the initiating factor in periodontitis, it has been well accepted that immune disorders further worsen the situation and non-resolving inflammation is consequently developed, which is the main cause of periodontitis-related tissue damage [3, 6]. Therefore, the traditional single antibacterial therapy may not be sufficient to solve such a complex problem of periodontitis [7]. Targeting the immune microenvironment could be a promising strategy for periodontitis [8].

Macrophages, a main regulator of the innate immune system, play a key role in controlling the inflammation process [9]. It has been revealed that targeting macrophages is a potential therapeutic intervention for periodontitis [10, 11]. For example, the repolarization of macrophages from M1-like inflammatory phenotype to M2-like anti-inflammatory phenotype can effectively ameliorate periodontitis [8].

Resveratrol (RSV) is a natural nonflavonoid polyphenol derived from several plant species and has anti-inflammatory and antioxidant effects [12]. For instance, resveratrol could regulate macrophage polarization to inhibit the inflammatory mediators in myocardial infarction [13] and acute gouty arthritis [14]. However, the therapeutic use of resveratrol is restricted due to its poor stability and water-solubility as well as the low bioavailability [15]. Therefore, an effective delivery system of resveratrol is important for its clinical application.

Nano-drug delivery systems can be used to improve the efficacy of drugs on macrophage reprogramming [16]. In this work, a macrophage-repolarization nanotherapeutic strategy was investigated for remodeling the immune microenvironment of periodontitis. The resveratrol liposomes were developed for local administration to the periodontal pocket to treat periodontitis. Liposomal formulations are among the most druggable nanocarriers, and thus naturally considered a priority in the study due to the translation potential.

## Materials and methods

### Materials, cells, and animals

Resveratrol and ibuprofen was purchased from MeilunBio (Dalian, China). Minocycline Hydrochloride was purchased from Sunstar Co., Ltd (Takatsuki, Japan). Dulbecco’s modified Eagle’s Medium (DMEM) and fetal bovine serum (Gibco®) were obtained from Thermo Fisher (Waltham, USA). The cocktail protease inhibitor, lipopolysaccharide, and Pluronic® F127 were purchased from Sigma-Aldrich (St. Louis, USA). *P.g*-LPS was from InvivoGen (San Diego, USA). Recombinant murine macrophage colony-stimulating factor (M-CSF), interferon-gamma (IFN γ), and interleukin-4 (IL-4) were purchased from Peprotech (Rocky Hill, USA). The qPCR SYBR® Green Master Mix and cDNA synthesis SuperMix kits were from Yeasen Biotech (Shanghai, China). The primers of IL-1β, IL-6, TNF-α, IL-12, IL-10, CD206, Arg-1, Chil3, TGF-β, CD163, CD86, iNOS, CCR7, and GAPDH were provided by Sango Biotech (Shanghai, China). Murine TNF-α, IL-1β, IL-6, IL-12, and IL-10 ELISA kits were from Lianke Biotech (Hangzhou, China). BCA protein assay kit, horseradish peroxidase (HRP)-conjugated goat anti-rabbit/mouse lgG secondary antibody, and crystal violet were from Beyotime (Shanghai, China). Coumarin 6 and were from J&K (Beijing, China). PC-98 T, cholesterol, and DSPE-PEG_2000_ were obtained from AVT (Shanghai, China). Artificial saliva (ISO/TR10271, pH 6.8–7) was purchased from Yuanye Biotech (Shanghai, China).

The bone marrow-derived macrophages (BMDM) were obtained from bone marrow-derived monocytes from Balb/c mice according to a standard protocol [17]. Murine macrophage cells (RAW264.7), mouse fibroblast cells (L929), and human vascular endothelial cells (HUVEC) were cultured in DMEM supplemented with 1% penicillin–streptomycin and 10% FBS in 5% CO_2_ at 37 °C. The M1Φ differentiation was induced using LPS (100 ng/mL) and IFN-γ (20 ng/mL), and M2Φ was induced using IL-4 (40 ng/mL). *Streptococcus mutans* (*S. mutans*) UA159, kindly provided by the Oral Microbiota and Systemic Disease Laboratory (Shanghai, China), were cultured anaerobically in the brain heart infusion broth (BHIB; BD, Franklin Lakes, USA) at 37 °C.

The BALB/c female mice (7–8 weeks old) were housed in an SPF facility. The animal experiments were performed according to the protocol (IACUC: 2020–06-HYZ-86) approved by the Ethics Committee of Shanghai Institute of Materia Medica, Chinese Academy of Sciences.

### Preparation of Lipo-RSV

The liposomes were prepared using a standard thin-film hydration method [18]. In brief, PT-98T, cholesterol, and DSPE-PEG_2000_ (20: 2: 1, w/w) were dissolved in chloroform and resveratrol in methanol. Resveratrol and phospholipids were mixed at a mass ratio of 1: 20 and the final ratio of the mixture solutions was chloroform: methanol = 20: 1 (v/v). The organic solvent was removed using rotary evaporation for 1 h at 40 °C. The thus-formed thin film was hydrated using PBS (pH 7) for 30 min at room temperature, followed by probe sonication for 5 min at 10 W. The thus-formed liposomes were extruded 21 times through polycarbonate membranes with a 200-nm pore size by a hand held extruder (Avestin, Ottawa, Canada) and then purified using a Sephadex G-50 column (GE Healthcare, Boston, MA, USA) to remove free resveratrol.

The prepared liposomes were mixed with Pluronic® F127 (20% w/w, 20 °C) to obtain a homogeneous liposomal dispersion before the animal experiment.

### Characterization of liposomes

The morphology of the Lipo-RSV was characterized by a cryo-electron microscope (Cryo-EM, FEI Talos Arctica G2, Thermo Fisher, Waltham, USA) operated at 200 kV. The zeta potential and particle size of the Lipo-RSV were measured by Zetasizer (Nano-ZS90, Malvern Instruments, UK).

The size, PDI, and zeta potential of Lipo-RSV were monitored at 37 °C and 4 °C in artificial saliva. The measurement procedure was described as in our previous report [18].

The drug-loading capacity (DL%) and encapsulation efficiency (EE%) of Lipo-RSV were determined by HPLC (1260 Infinity, Agilent technologies, USA) after purification by a Sephadex G100 column (GE Healthcare, Chicago, USA). The chromatographic conditions were described as follows: Agilent-C18 column (250 × 4.6 mm, 5 μm);mobile phase, 0.1% phosphoric acid in water/acetonitrile (60: 40, *v/v*); flow rate of 1 ml/min; detection wavelength, 375 nm.

Lipo-RSV was dialyzed (MWCO 14 K) against 50 ml PBS (pH 6.8, containing 0.2% Tween-80) to maintain sink condition. It was stirred at 37 °C and a speed of 150 rpm. At the predetermined time intervals, 0.2 ml of release medium was withdrawn and then replenished with 0.2 ml fresh release medium. The amount of resveratrol released from Lipo-RSV was determined by HPLC.

### Uptake study in macrophage

The BMDMs were seeded in the 24-well plates at a density of 5 × 10^4^ cells per well and induced to the M1-like phenotype as described above. On the following day, M1Φ were incubated with the coumarin-6-labeled liposomes (200 ng/ml) for 2 h and then washed three times with PBS. The cells were fixed with paraformaldehyde (4%) for 15 min and stained with DAPI (1 μg/ml) for 10 min. The cells were observed by fluorescent microscopy.

### In vitro cytotoxicity study

The RAW264.7, HUVEC, or L929 were seeded into the 96-well plates at a density of 5 × 10^3^ per well, respectively. After 24 h, the cells were incubated with RSV or Lipo-RSV in different concentrations for 24 h. Then 10 μl CCK8 (diluted by fresh DMEM) was added to each well. The absorbance was detected by a microplate reader at 450 nm. In addition, the cells were also seeded into the 24-well plates and treated with RSV (15 μΜ)or Lipo-RSV (15 μM). When growing to 60% confluence, the cells were then incubated with a medium containing calcein and propidium iodide for 30 min, washed with PBS, and observed under a fluorescence microscope.

### Effects of Lipo-RSV on macrophage polarization and inflammatory responses

After the BMDMs and the mouse gingival tissues were collected, RNA was extracted by adding the appropriate amount of TRIzol reagent (Tiangen, Beijing, China) according to the manufacturer’s instruction, and then the reverse-transcribed cDNA was analyzed. The reverse transcription conditions were as described in a previous report [18], and the primer sequence is listed in Additional file 1: Table S1. The MΦ phenotype was detected by flow cytometry (FCM) and Western blotting, in which anti-CD206 was used to label the M2-like MΦ and anti-CD86 to label the M1-type MΦ. Besides, the levels of cytokines secreted by the BMDM and in the gingival tissues were detected by the ELISA kits. The antibody information is listed in Additional file 1: Tables S2, S3, and S4.

### ROS scavenging activity assay

RSV or Lipo-RSV in a conditioned medium with 1 μg/ml of LPS was added to the BMDMs for 2 h incubation. The BMDMs were then incubated with DCFH-DA (Beyotime, Shanghai, China) for 30 min, and subjected to flow cytometric measurement and fluorescence microscopy.

### Bacteria antibiotic susceptibility assay in vitro

RSV and Lipo-RSV at a final concentration of 15 μM were added to bacterial suspension (1 × 10^6^ CFU/ml) and incubated at 37 °C for 6 h. After diluted 1,000 times, 50 μl of the bacteria suspension was applied onto agar plates and incubated at 37 °C for 24 h.

### Therapeutic efficacy in vivo

The ligature-induced periodontitis model was developed by ligation with 5–0 silk into the subgingival and injected with 10 μl *P.g*-LPS (1 mg/ml) for 2 weeks [19]; the model was used to estimate the therapeutic effects of Lipo-RSV. Briefly, the model animals were randomly divided into 4 groups (N = 4/group). The groups were given 10 μl PBS, 4 μg resveratrol, 4 μg Lipo-RSV, or 2 μg minocycline hydrochloride, respectively, in the gingival sulcus every other day. Meanwhile, a control group was set up using the healthy mice. After 2 weeks, the gingiva and upper jaw were harvested for histology analysis (H&E), methylene blue staining, and immunofluorescence[8]. At the experimental endpoint, the histology examination of the major organs was performed and no pathological changes were found (Additional file 1: Figure S10).

The gingiva tissues of the mice were collected, and lysed by RIPA buffer with cocktail protease inhibitors and tissue homogenizer. The protein level of IL-1β, IL-6, TNF-α, p65, p-p65, NLRP3, COX2, Caspase1, CD206, iNOS, CD86, STAT1, STAT3, p-STAT1, and p-STAT3 in the gingiva were detected by a standard Western blotting protocol. The protein expression was analyzed by ImageJ (n = 3). In addition, qPCR assay was also performed to detect the mRNA expression of *IL-1β, IL-6, TNF-α, CD86, CD163, Arg-1,* and *TGF-β* in the gingiva tissues (n = 3).

In another animal experiment, the periodontitis model mice were treated with an anti-inflammatory drug Ibuprofen (100 μg/mouse) to compare with Lipo-RSV therapy following a procedure described above.

### Statistical analysis

The data are expressed as the mean ± standard deviation. All data are analyzed by unpaired Student’s T-test (between two groups) or one-way ANOVA (between multiple groups), n ≥ 3. *P < 0.05, **P < 0.01, ***P < 0.001, ****P < 0.0001.

## Results

### Characterizations of Resveratrol-loaded liposome

**Fig. 1:**
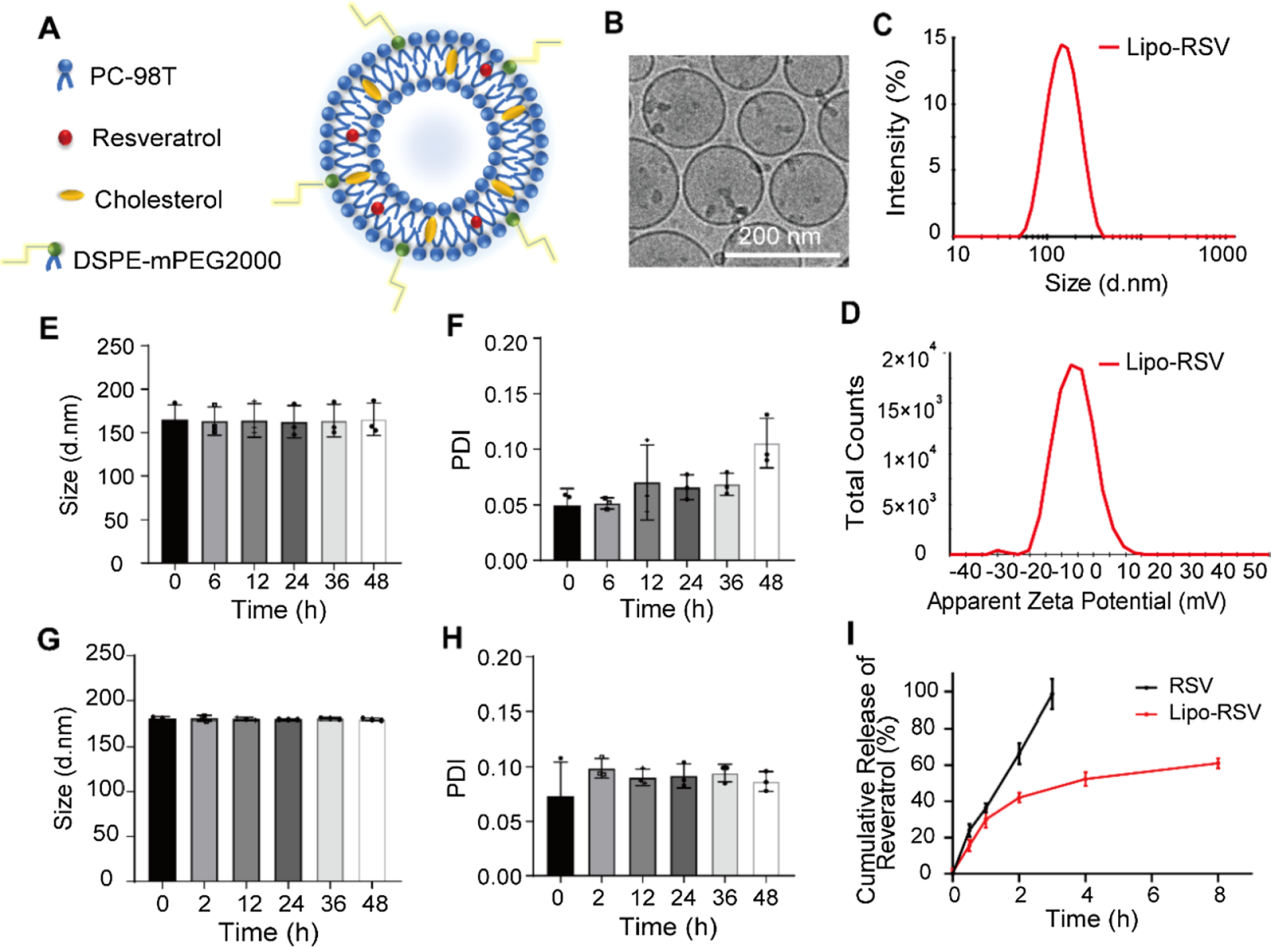
Characterization of Lipo-RSV. **A** Schematic illustration of Lipo-RSV. **B** Cryo-EM of Lipo-RSV. **C** Size distribution of Lipo-RSV. **D** ζ potential measurement. **E**, **F** Stability of Lipo-RSV in artificial saliva (4 °C). **G**, **H** Stability of Lipo-RSV in artificial saliva (37 °C). **I** In vitro release of resveratrol from Lipo-RSV

The liposomes were prepared using a standard thin-film hydration method (Fig. 1A). Cryo-EM images show the morphology of Lipo-RSV in a regular shape within 200 nm (Fig. 1B). The mean size of the Lipo-RSV was about 136 nm, and the zeta potential was –11 mV (Additional file 1: Table S5). The characterization results of Lipo-RSV in 20% Pluronic F127 were shown in Additional file 1: Table S5 and Figure S1. Lipo-RSV was stable in 20% Pluronic F127.

The drug-loading capacity (DL%) and encapsulation efficiency (EE%) of Lipo-RSV were measured to be 81.3% and 3.9% (Additional file 1: Table S6).

The Lipo-RSV remained stable in the artificial saliva at 37 °C (mimicking the oral cavity temperature) or at 4 °C (storage conditions); there were just minor changes in size (Fig. 1E, 1G, H), suggesting the stability of Lipo-RSV. In artificial saliva, the Lipo-RSV exhibited a sustained release effect, and around 60% of the drug was released in 8 h (Fig. 1I). Notably, Lipo-RSV acted in the inflammatory macrophages that are rich in the pathological site of periodontitis. Once the locally administered liposomes are taken up by the macrophages, the released drug will induce the macrophage repolarization that occurs within 24 h according to a routine in-vitro induction protocol [20].

### Cellular uptake and cytotoxicity studies

After incubation with the coumarin 6-labeled liposomes, the BMDMs were observed under a fluorescence microscope and the results showed a high uptake efficiency in the BMDMs within 2 h (Additional file 1: Figure S2A).

At a dose of 15 μM, both RSV and Lipo-RSV showed little cytotoxicity in the HUVEC, RAW264.7, and L929 cells (Additional file 1: Figure S2B–D), measured by calcein/PI cell viability assay. Moreover, even when a dose was increased to 100 μΜ, all the cells retained 80% viability (Additional file 1: Figure S2E–J), determined by the CCK8 assay. It demonstrated that Lipo-RSV had good biocompatibility to these cells at the designated concentrations.

### Lipo- RSV drives macrophage polarization from pro-inflammatory to anti-inflammatory phenotype

The active M1 macrophages were treated with different concentrations of resveratrol. The qRT-PCR results showed that resveratrol up-regulated the M2-related genes (e.g., CD206 and IL-10) and downregulated the M1-related genes (e.g., iNOS and IL-1β) at the concentration of 15 μM (Additional file 1: Figure S3).

**Fig. 2:**
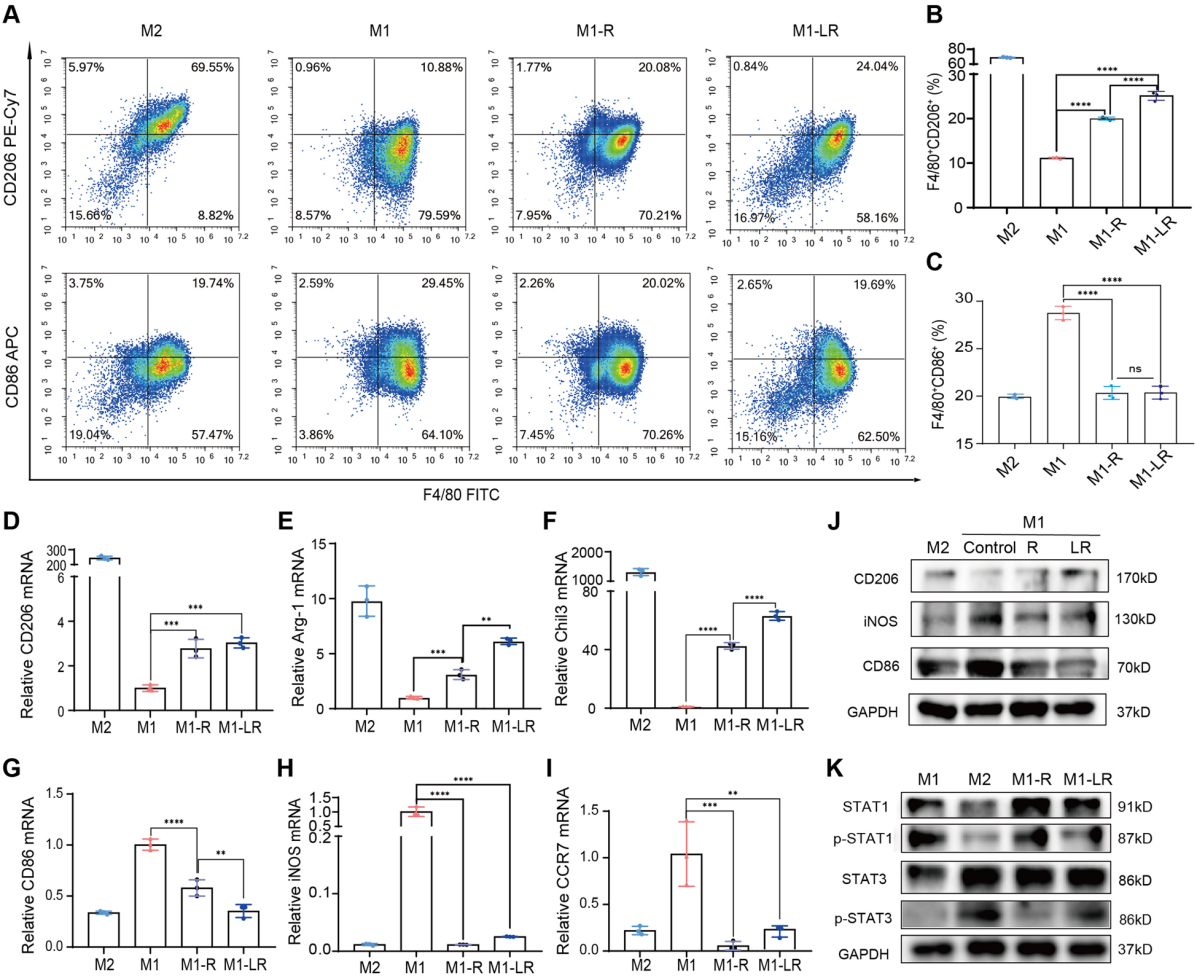
Lipo-RSV promoted macrophage repolarization from M1- to M2-like phenotype. **A** Flow cytometry analysis of the M1-like subset (F4/80^+^CD86^+^) and M2-like subset (F4/80^+^CD206^+^). Statistical analysis of the percent of **B** M1Φ (F4/80^+^CD206^+^) or **C** M2Φ (F4/80^+^CD86^+^).The mRNA leavel of **D**–**F** M2-related markers (CD206, Arg-1 and Chil3), and **G**–**I** M1 macrophage markers (CD86, iNOS and CCR7) in the activated macrophages. **J** Lipo-RSV upregulate CD206 and downregulate iNOS and CD86 protein. **K** Lipo-RSV promoted the phosphorylation of STAT3 and inhibited the phosphorylation of STAT1. Data are presented as mean ± SD (n = 3); ns, no significance, **P < 0.01, ***P < 0.001, ****P < 0.0001. R: RSV (15 μM); LR: Lipo-RSV (equal to 15 μM RSV)

The qRT-PCR results revealed the re-polarization effect of Lipo-RSV. The mRNA levels of M2 macrophage markers (CD206, Arg-1, and Chil3) were up-regulated (Fig. 2D–F), and the M1 macrophage markers (CD86, iNOS, and CCR7) were down-regulated (Fig. 2G–I). Lipo-RSV inhibited the phosphorylation of STAT1 but promoted the phosphorylation of STAT3 (Fig. 2K). Therefore, it demonstrated Lipo-RSV can repolarize the macrophages from M1 to M2 phenotype through regulating p-STAT1 and p-STAT3.

After resveratrol and Lipo-RSV treatment, the morphology of macrophages showed shuttle-shaped (Additional file 1: Figure S4), which is a characteristic of M2 macrophages.

The flow cytometric analysis showed Lipo-RSV treatment increased the percentage of F4/80^+^CD206^+^ macrophages by 14% (Fig. 2B) while F4/80^+^CD86^+^ macrophages were decreased by 8% (Fig. 2C). What is more, the ratio of M2/M1 was three times higher than that of untreated M1Φ (Additional file 1: Figure S5).

### Anti-inflammatory effect and ROS scavenging capability of Lipo-RSV in vitro

**Fig. 3:**
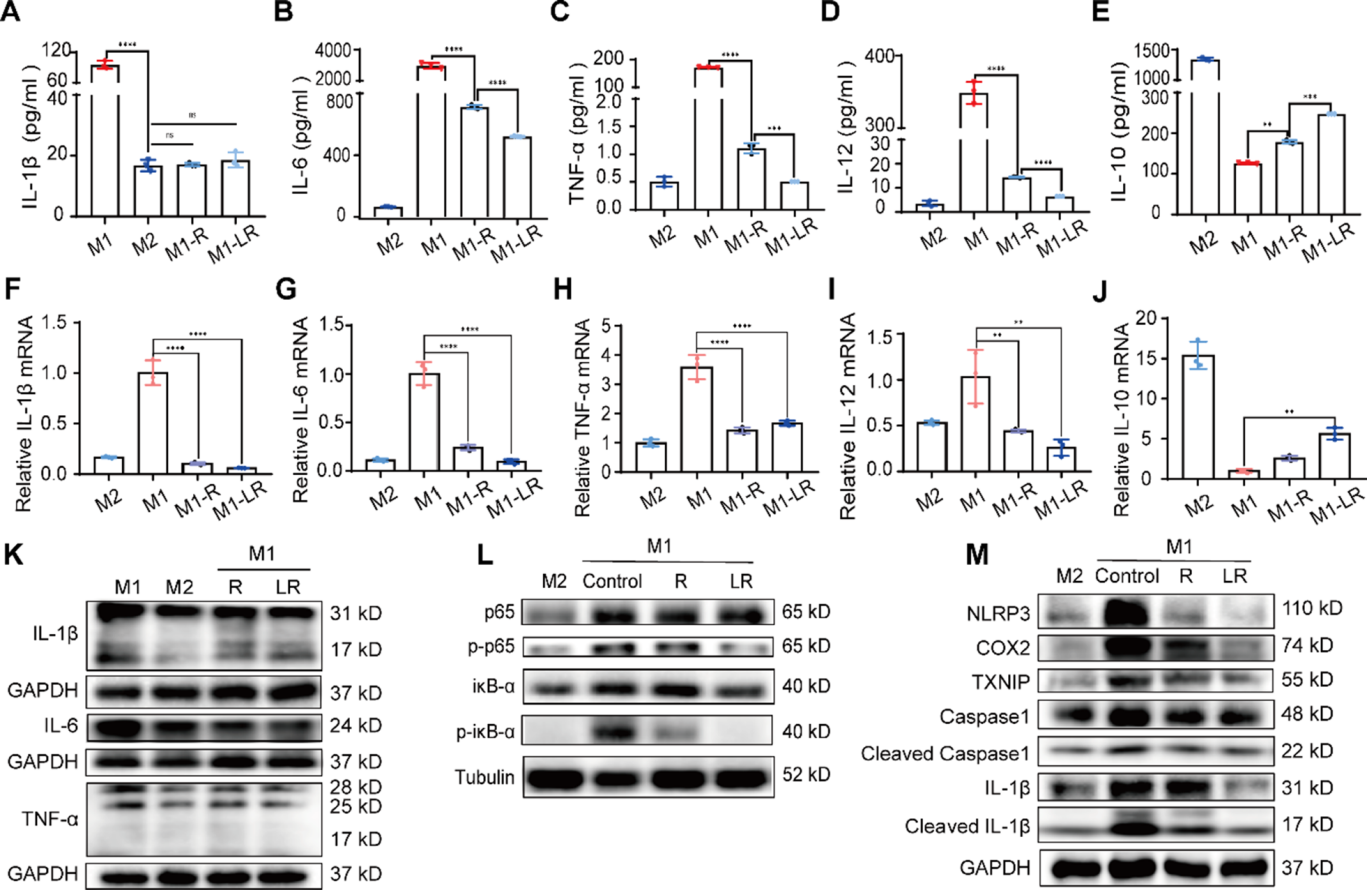
Anti-inflammatory effect of Lipo-RSV in vitro. **A**–**E** ELISA analysis of the levels of IL-1β, IL-6, TNF-α, IL-12, and IL-10. **F**–**J** mRNA levels of IL-1β, IL-6, TNF-α, IL-12, and IL-10 by qRT-PCR. **K** The down-regulation of IL-1β, IL-6, and TNF-α in M1Φ after treatment. **L** Expression of the NF-κB pathway-related proteins after treatment. **M** Expression of the inflammasome-related proteins after treatment. Data are presented as mean ± SD (n = 3); ns, no significance, **P < 0.01, ***P < 0.001, ****P < 0.0001. R: RSV (15 μM); LR: Lipo-RSV ( equal to 15 μM RSV)

After Lipo-RSV treatment for 24 h, the secreted pro-inflammatory cytokines (IL-1β, IL-6, TNF-α, and IL-12) by the M1Φ were attenuated (Fig. 3A–D). By contrast, the anti-inflammatory IL-10 was up-regulated (Fig. 3E). The transcriptional level also verified the same trend (Fig. 3F–J). Similarly, the Western blotting results also demonstrated the decrease of the pro-inflammatory cytokines of IL-1β, IL-6, and TNF-α (Fig. 3K). The effects were associated with the inhibition of the NF-κB signaling (Fig. 3L) and suppression of the inflammasome activation (Fig. 3M).

**Fig. 4:**
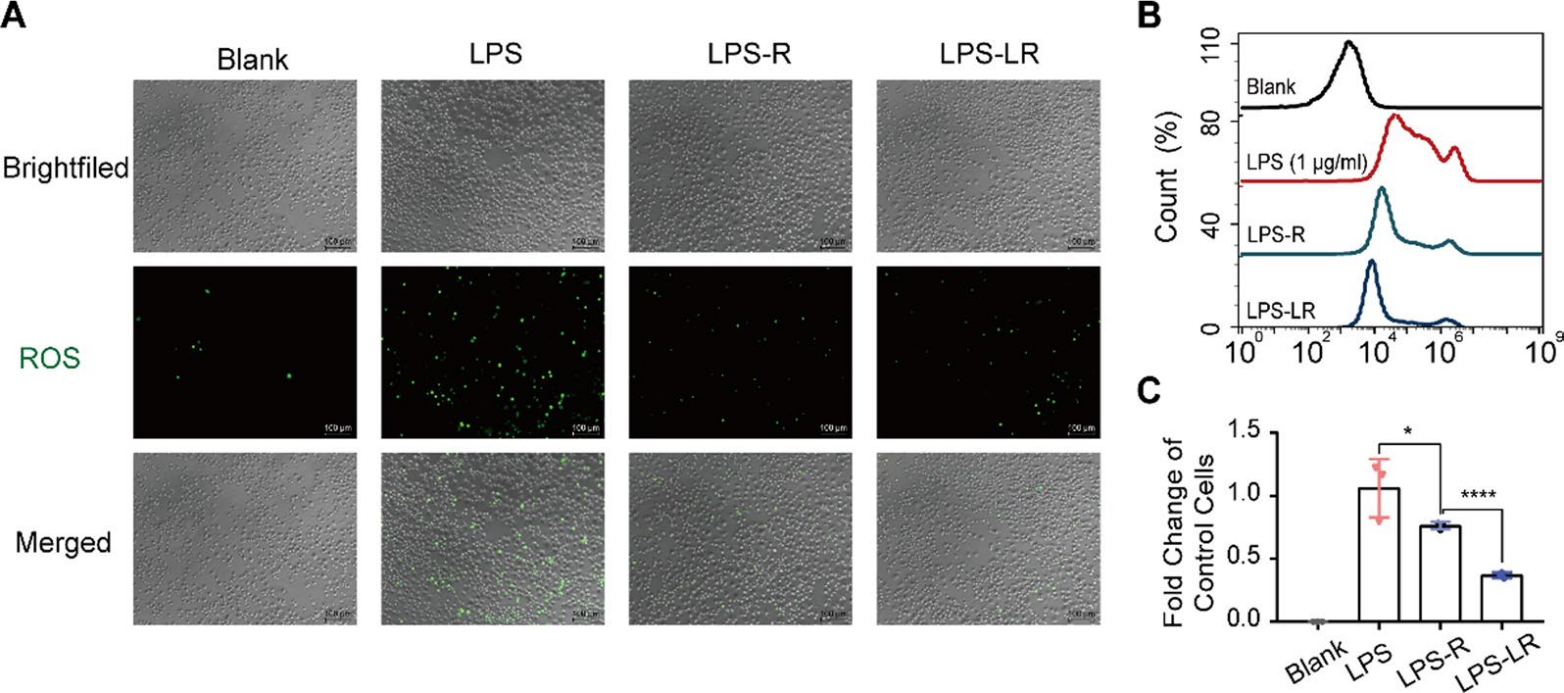
ROS scavenging effect. **A** Fluorescence microscopy shows the clearance of ROS after treatment with Lipo-RSV. **B** The reduced intracellular ROS levels in M1Φ after treatment. **C** Statistical analysis of ROS levels. Data are presented as mean ± SD (n = 3); ns, no significance, *P < 0.05, ****P < 0.0001. LPS: 1 μg/ml; LPS-R: 1 μg/ml LPS + 15 μM RSV; LPS-LR: 1 μg/ml LPS + 15 μM Lipo-RSV

The ROS level in the M1Φ (stimulated by LPS, 1 μg/ml) was high, but it decreased after treatment with Lipo-RSV (Fig. 4A, B). The results demonstrated that resveratrol-based treatment decreased the ROS level in the M1Φ, and Lipo-RSV exhibited a better effect than free resveratrol (Fig. 4C).

### Lipo-RSV ameliorates periodontitis

**Fig. 5:**
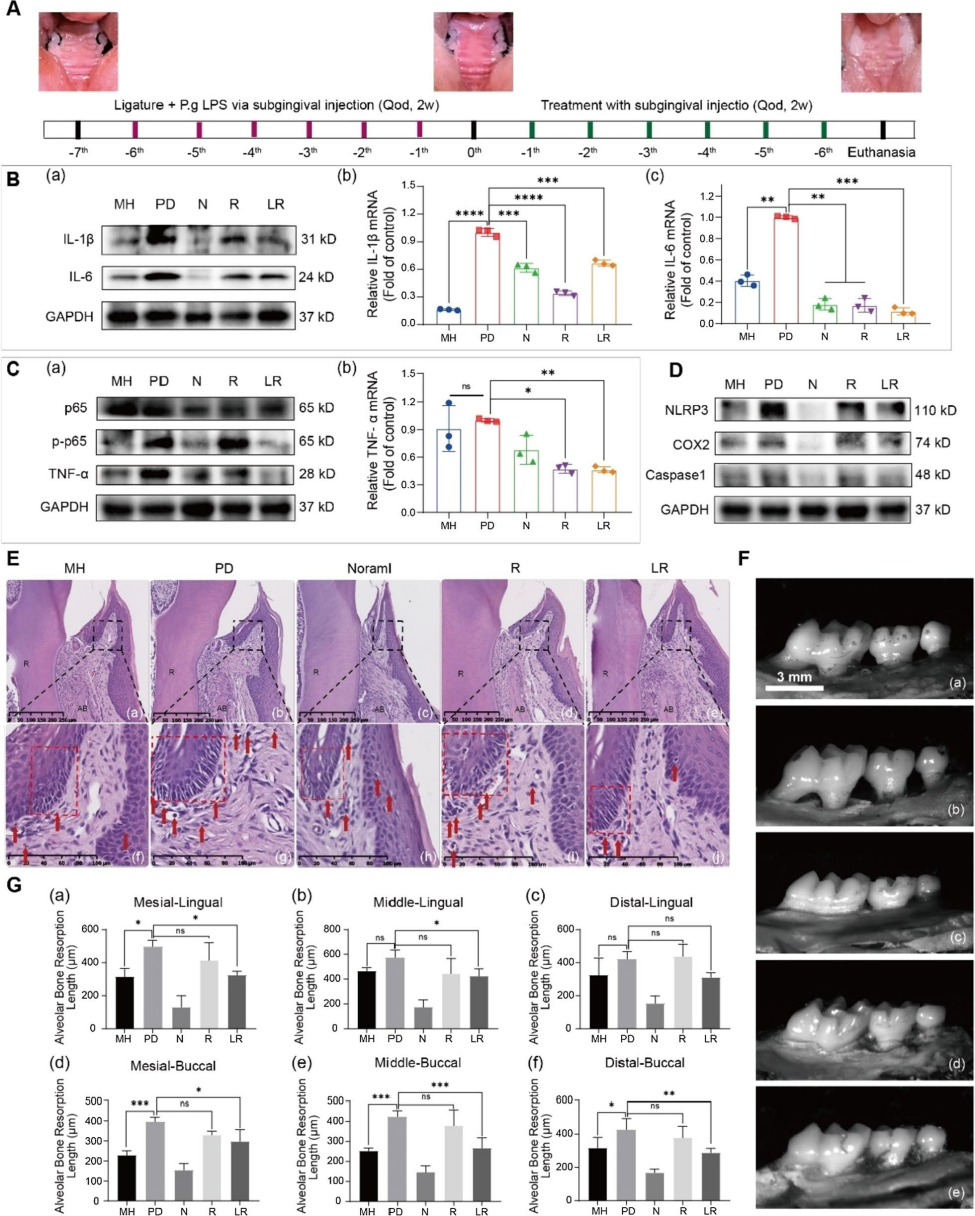
Lipo-RSV ameliorates Periodontitis. **A** Schematic illustration of P.g LPS-induced periodontitis in Balb/c mice and the treatment regimen. **B** IL-1β and IL-6 expression in the gingival after treatment. (a) Western blotting assay of IL-1β and IL-6 level in the gingiva, (b) The IL-1βand IL-6 levels of the gingiva were qualified by qRT-PCR assay (n = 3). **C** p65, p-p65, and TNF-α expression in the gingival after treatment. (a) Western blotting results, (b) The TNF-α levels of the gingiva were qualified by qRT-PCR assay (n = 3). **D** NLRP3, COX2, and caspase1 expression in the gingival after treatment. **E** HE staining of the gingiva. Red dashed boxes indicate epithelial junctions, and red arrows indicate infiltrating inflammatory cells. R: dental root; AB: alveolar bone. **F** Representative figures as indicated by alveolar bone loss and root exposure examined by methylene blue staining. **a** MH: minocycline hydrochloride, **b** PD: periodontitis, **c** N: normal, **d** R: RSV, **e** LR: Lipo-RSV. **G** Statistical analysis of the alveolar bone resorption length. Data are presented as mean ± SD (n = 3); ns, no significance, *P < 0.05, **P < 0.01, ***P < 0.001, ****P < 0.0001

An in vivo study was conducted to further confirm the effects of Lipo-RSV on periodontitis (Diagram Fig. 5A). The animal results indicated that, after two weeks of treatment, Lipo-RSV effectively ameliorated periodontitis, evidenced by a decrease in the expression of inflammatory cytokines in the gingival tissue (Fig. 5B, C) and down-regulation of the essential proteins of NF-κB signaling (Fig. 5C) and inhibition of NLRP3 inflammatory signaling (Fig. 5D).

Furthermore, the H&E staining showed the normalization of periodontal tissue structure after Lipo-RSV treatment (Fig. 5Ea-e). It revealed that the repair of the epithelial barrier was accompanied by the decreased number of spongiosis (indicated by the red dashed box in Fig. 5Ef–j) and the reduced inflammatory cells in the connective tissue layer (indicated by the red arrow in Fig. 5Ef–j) after Lipo-RSV treatment. The methylene blue staining of alveolar bone showed that Lipo-RSV effectively reduced the alveolar bone resorption in the lingual and buccal tissues (Fig. 5F, G).

**Fig. 6:**
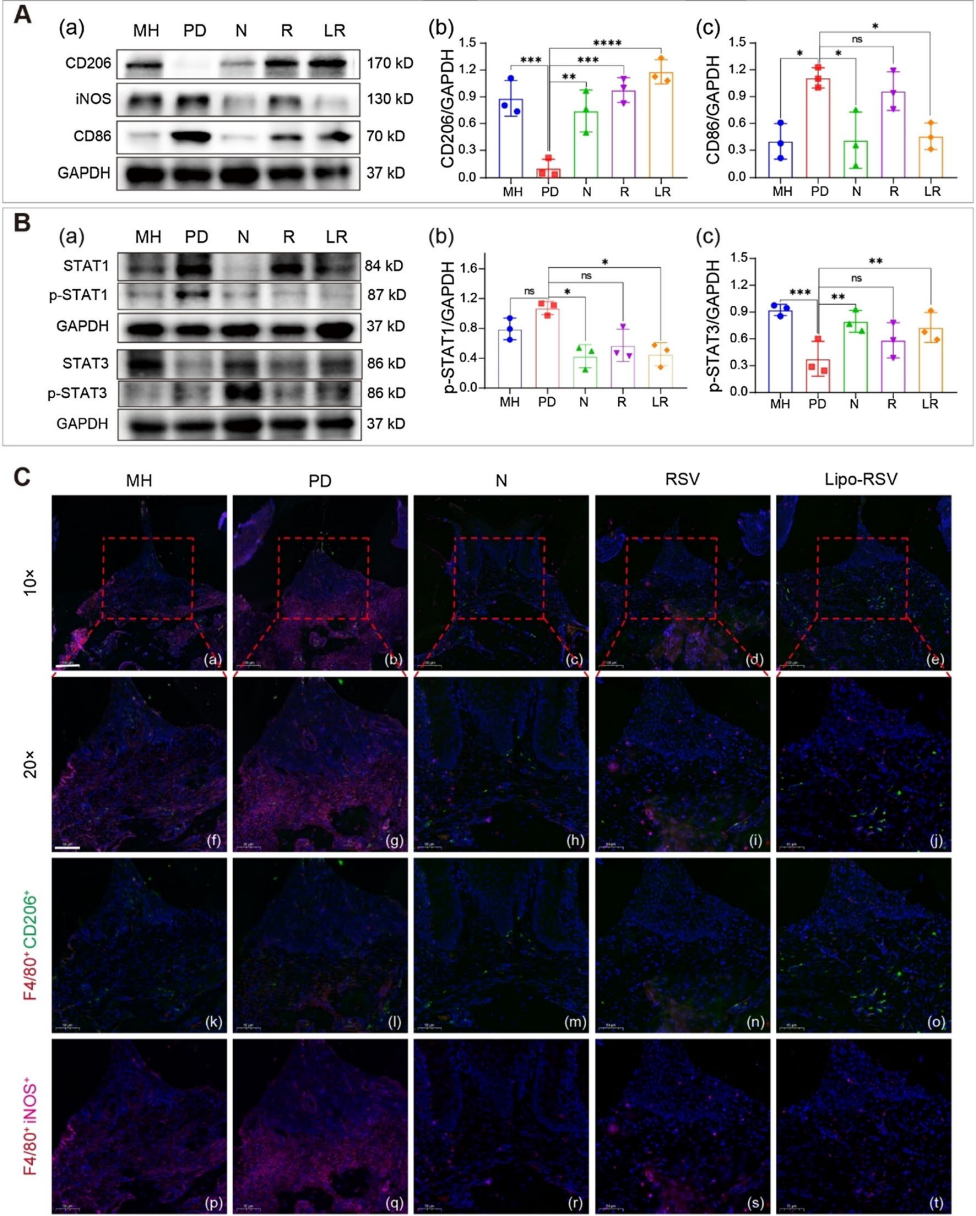
Identification of macrophages in periodontitis-associated soft tissue lesions. **A** (a) Western blotting results of CD206, iNOS, and CD86 expression in the gingival tissues. (b, c) The CD206 and CD86 levels in gingiva were qualified analyzed by WB and ImageJ (n = 3). **B** (a) Western blotting results of the level of p-STAT1 and p-STAT3. (b, c) The p-STAT1 and p-STAT3 levels in gingiva were qualified analyzed by WB and ImageJ (n = 3). **C** Macrophages stained for F4/80 (Cy3), CD206 (AF488), iNOS (Cy5), and cell nuclear (DAPI). Scale bar 10 μm (a–e), scale bar 50 μm (f–t). R: RSV, LR: Lipo-RSV. Data are presented as mean ± SD (n = 3); ns, no significance, *P < 0.05, **P < 0.01, ***P < 0.001, ****P < 0.0001

The Western blotting result showed the increased expression of CD206 (M2Φ maker) and the decreased expression of iNOS and CD86 (M1Φ makers) in the gingiva of the periodontitis mice after treatment with Lipo-RSV (Fig. 6A). At the genetic level, *CD86* was decreased and M2-biomarkers (*CD163, Arg-1,* and *TGF-β*) were increased after treatment (Additional file 1: Figure S6). The immunofluorescence results also displayed the increased expression of CD206 and reduced expression of iNOS in M1Φ (F4/80^+^) in the gingival tissue after Lipo-RSV treatment (Fig. 6C). The expression of p-STAT3 was increased and p-STAT1 was suppressed in the gingival tissues from the Lipo-RSV-treated mice (Fig. 6B), with a similar trend to the in vitro results.

Antibiotic treatment (e.g., minocycline) is still the mainstream regimen in periodontitis [21], though its obvious drawbacks. However, periodontitis is a complicated disease involving multiple causes such as immune imbalance, bacterial activity, and osteogenesis. To further demonstrate the Lipo-RSV efficacy, it was also compared with an anti-inflammatory drug, ibuprofen. The results also revealed that Lipo-RSV had similar anti-bone resorption activity as ibuprofen (Additional file 1: Figure S7) and its anti-inflammatory effect was comparable to ibuprofen (Additional file 1: Figure S8).

It should be noted that RSV also has antibacterial activity [12]. Our results also revealed Lipo-RSV can efficiently inhibit Streptococcus mutans, a dental disease-related pathogen (Additional file 1: Figure S9). Therefore, Lipo-RSV treatment could involve multiple mechanisms.

## Discussion

Minocycline is a typical antibacterial drug used in periodontitis treatment. Notably, it also has an anti-inflammatory effect [22]. The treatment efficacy of Lipo-RSV was similar to that in the minocycline-treated mice. In addition, we also compared the efficacy of Lipo-RSV with a commonly used anti-inflammatory drug ibuprofen, and the result showed similar efficacy. It implied that Lipo-RSV could be potentially used for treating periodontitis via immunomodulation.

Indeed, the oral cavity is a bacteria-rich environment, but normally these bacteria do not cause diseases. Only under a circumstance of host immune dysfunctions, periodontitis occurs and eventually develops into non-resolving inflammation that is no longer driven by bacterial infection. Therefore, in our study, we placed a focus on remodeling the immune microenvironment as a treatment strategy.

Macrophages play a critical role in orchestrating the immune response to infection/inflammation [23]. Clinical studies revealed that an increased ratio of M1/M2 phenotypes of macrophages positively correlates with the degree of periodontal inflammation [24]. Because of the high plasticity of macrophages, targeting macrophages has been a cutting-edge strategy for many diseases related to immune imbalance [25].

The p-STAT1 and p-STAT3 pathways are associated with the macrophage polarization between M1 and M2 phenotypes [13, 26, 27]. It has been reported that RSV has an activity of regulating macrophages through the JAK2-SATA3 pathway [13]. However, RSV is considered to be poorly druggable because of its low water solubility and stability. This limits its therapeutical application. Therefore, we developed a Lipo-RSV delivery system for reprogramming macrophages from M1 to M2 to serve as an antibiotic-free strategy for treating periodontitis.

ROS is a nexus of cellular homeostasis [28, 29]. Increasing evidence showed that ROS-regulated therapy is useful for modulating the immune microenvironment [30, 31]. RSV is a potent ROS scavenger that protects against lipid peroxidation in cell membranes and DNA damage and thus promotes wound healing [32, 33]. Notably, Lipo-RSV could eliminate about 50% of ROS in M1 macrophages, and decrease the expression of NLRP3, caspase1, and IL-1β. The excessive ROS production by inflammatory macrophages interferes with cell cycle progression and causes irreversible damage to periodontal tissues [13, 34]. ROS also plays an essential role in maintaining the inflammatory phenotype of macrophages via activating NLRP3 inflammasome and promoting IL-1β induction [8, 35, 36]. Therefore, ROS clearance could be an important mechanism for Lipo-RSV for remodeling the immune microenvironment. It is not fully explicated the molecular mechanisms of ROS regulating the inflammatory signaling; it is likely related to the mitochondrial state [37, 38].

Nanomedicine has been widely explored for its targeting delivery functions and to improve the treatment outcomes [39–41]. In addition, nanomedicine in immunoregulation has attracted great attention [42], and local administration of nanomedicine has been demonstrated for the therapeutic benefits of direct access of drugs to the targeted tissue [7, 43, 44]. The locally administered Lipo-RSV was developed for treating periodontitis via remodeling the immune microenvironment. The liposomal formulation can enhance the solubility and stability of RSV. Due to the phagocytosis nature of the inflammatory macrophages, Lipo-RSV could be beneficial for targeting macrophages. As evidence, our results showed that Lipo-RSV had superior effects on macrophage repolarization than free RSV.

## Conclusion

In summary, we developed a Lipo-RSV system to treat periodontitis by modulating p-STAT1 and p-STAT3 to reprogram the macrophages from M1- to M2-like phenotype. The treatment can effectively suppress the inflammation, evidenced by the reduced secretion of pro-inflammatory cytokines (IL-1β, IL-6, TNF-α, and IL-12) and the increased anti-inflammatory factor IL-10 via inhibiting the NF-κB/NLRP3 signaling pathways. Given these, Lipo-RSV provides a promising antibiotic-free treatment method for periodontitis management and has translation potential.

## Supplementary Information

**Additional file 1.** Additional Tables S1–S5 and Figures S1–S9.

## Data Availability

They are included in the result and method sections.

## Abbreviations

RSV: Resveratrol
Lipo-RSV: Resveratrol-loaded liposomes
MH: Minocycline hydrochloride
PD: Periodontitis
M1Φ: M1-like phenotype macrophage
M2Φ: M2-like phenotype macrophage
p-STAT3: Phosphorylation of signal transducer and activator of transcription 3
p-STAT1: Phosphorylation of signal transducer and activator of transcription 1
ROS: Reactive oxygen species
NF-κB: Nuclear factor kappa-B
iκB-α: Inhibitor of nuclear factor kappa-B
p-iκB-α: Phospho of IκBα
NLRP3: NOD-, LRR- and pyrin domain-containing 3
COX2: Cyclooxygenase-2
TXNIP: Thioredoxin-interacting protein
IL-1β: Interleukin-1beta
IL-6: Interleukin-6
IL-10: Interleukin-10
IL-12: Interleukin-12
TNF-α: Tumor necrosis factor-α
*P.g*: Porphyromonas gingivalis
LPS: Lipopolysaccharide
Arg-1: Arginase-1
Chil3: Chitinase-like 3
iNOS: Inducible nitric oxide synthase
CCR7: CC motif chemokine receptor 7
PC-98T: Egg yolk lecithin (PC-98T)
DSPE-PEG2000: 1,2-Distearoyl-sn-glycero-3-phosphoethanolamine-*N*-[(polyethylene glycol)-2000]
Cryo-EM: Cryo-electron microscope
TEM: Transmission electron microscopy
