# Supplementary material for: Remodeling immune microenvironment in periodontitis using resveratrol liposomes as an antibiotic-free therapeutic strategy
Source: J Nanobiotechnology. 2021 Dec 20;19:429. doi: 10.1186/s12951-021-01175-x (PMC8686397; doi:10.1186/s12951-021-01175-x)

**Additional Materials**

Remodeling Immune Microenvironment in Periodontitis Using Resveratrol Liposomes as an antibiotic-free therapeutic strategy

*Junyu Shi^1#^, Yi Zhang^1#, 2^, Xiaomeng Zhang^1^, Jianxu Wei^1^, Ruiying Chen^1^, Jiazheng Hou ^2^, Bing Wang^2^, Hongchang Lai ^1,^*, Yongzhuo Huang^2, 3, 4,^**

**[Table S1](#正文tableS1)** Primer sequences are used for real-time PCR.

| Gene | Forward Primer (5′ to 3′) | Reverse Primer (5′ to 3′) |
| --- | --- | --- |
| CD206 | TCTTTGCCTTTCCCAGTCTCC | TGACACCCAGCGGAATTTC |
| Arg-1 | GACCACAGTCTGGCAGTTGG | CGTTGAGTTCCGAAGCAAGC |
| Chil3 | CAGGTCTGGCAATTCTTCTGAA | GTCTTGCTCATGTGTGTAAGTGA |
| TGF-β | CTTCAATACGTCAGACATTCGGG | GTAACGCCAGGAATTGTTGCTA |
| CD163 | ATGGGTGGACACAGAATGGTT | CAGGAGCGTTAGTGACAGCAG |
| CD86 | TTGTGTGTGTTCTGGAAACGGAG | AACTTAGAGGCTGTGTTGCTGGG |
| iNOS | ACATCGACCCGTCCACAGTAT | CAGAGGGGTAGGCTTGTCTC |
| CCR7 | TGTACGAGTCGGTGTGCTTC | GGTAGGTATCCGTCATGGTCTTG |
| GAPDH | ATGGGTGTGAACCACGAGA | CAGGGATGATGTTCTGGGCA |
| IL-1β | CTTCAGGCAGGCAGTATCACTC | TGCAGTTGTCTAATGGGAACGT |
| IL-6 | ACAACCACGGCCTTCCCTAC | TCTCATTTCCACGATTTCCCAG |
| IL-12 | ATGGAGTCATAGGCTCTGGAAA | CCGGAGTAATTTGGTGCTTCAC |
| TNF-α | CAGGCGGTGCCTATGTCTC | CGATCACCCCGAAGTTCAGTAG |
| IL-10 | GCTCTTACTGACTGGCATGAG | CGCAGCTCTAGGAGCATGTG |

[**Table S2**](#正文tableS2) **Dilutions for Primary Antibodies for Western Blotting**

| Antibody | Dilution | Supplier | Product No. |  |
| --- | --- | --- | --- | --- |
| Anti-CD206 | 1:1000 | Absin (Shanghai, China) | abs125294 | |
| Anti-CD86 | 1:1000 | Abways (Shanghai, China) | cy5238 | |
| Anti-iNOS | 1:1000 | Proteintech (Wuhan, China) | 18985-1-AP | |
| Anti-GAPDH | 1:20,000 | Proteintech (Wuhan, China) | 60004-1-lg | |
| Anti-alpha-Tubulin | 1:20,000 | Proteintech (Wuhan, China) | 11224-1-AP | |
| Anti-STAT3 | 1:1000 | Abways (Shanghai, China) | AB3283 | |
| Anti-phospho-STAT3 | 1:1000 | CST (Boston, USA) | 9145T | |
| Anti-STAT1 | 1:1000 | CST (Boston, USA) | 14994T | |
| Anti-phospho-STAT1 | 1:1000 | Abways (Shanghai, China) | cy5702 | |
| Anti-NLRP3 | 1:1000 | CST (Boston, USA) | 20836T | |
| Anti-TXNIP | 1:1000 | CST (Boston, USA) | 20836T | |
| Anti-COX2 | 1:1000 | CST (Boston, USA) | 12282S | |
| Anti-ASC | 1:1000 | CST (Boston, USA) | 20836T | |
| Anti-Caspase1 | 1:1000 | CST (Boston, USA) | 20836T | |
| Anti-Cleaved Caspase1 | 1:1000 | CST (Boston, USA) | 20836T | |
| Anti-IL-1β | 1:1000 | CST (Boston, USA) | 20836T | |
| Anti-Cleaved IL-1β | 1:1000 | CST (Boston, USA) | 20836T | |
| Anti-IL-6 | 1:1000 | CST (Boston, USA) | 12912S | |
| Anti-TNF-α | 1:1000 | CST (Boston, USA) | 11948T | |
| Anti-p65 | 1:1000 | CST (Boston, USA) | 6956T | |
| Anti-p-p65 | 1:1000 | CST (Boston, USA) | 3033S | |
| Anti-iκB-α | 1:1000 | CST (Boston, USA) | 4814T | |
| Anti- p-iκB-α | 1:1000 | CST (Boston, USA) | 2859T | |

[**Table S3**](#正文tableS3) **Antibodies for Flow Cytometry**

| **Specificity** | **Clone** | **Fluorochrome** | **Supplier** |
| --- | --- | --- | --- |
| F4/80 | BM8 | FITC | BioLegend (san Diego, CA) |
| CD206 | C068C2 | PE-Cy7 | BioLegend (san Diego, CA) |
| CD86 | GL-1 | APC | BioLegend (san Diego, CA) |

**[Table S4](#正文TableS4) Dilutions for Primary Antibodies for Immunohistochemical**

| **Antibody** | **Dilution** | **Supplier** | **Product number** |
| --- | --- | --- | --- |
| Anti-F4/80-Cy3 | 1:400 | CST | 70076S |
| Anti-CD206-AF488 | 1:200 | PTG | 18704-1-AP |
| Anti-iNOS-Cy5 | 1:100 | Bioss | BS-0162R |

**[Table S5](#正文tableS5) The particle size, PDI, the zeta potential of Lipo-RSV and Lipo-RSV in 20% Pluronic F127 (Lipo-RSV-F127)**

|  | **Particle size (nm)** | **PDI** | **ζ potential(mV)** |
| --- | --- | --- | --- |
| Lipo-RSV 1 | 135.6 | 0.11 | -14.6 |
| Lipo-RSV 2 | 135.9 | 0.059 | -11.1 |
| Lipo-RSV 3 | 136.8 | 0.080 | -8.6 |
| Mean | 136.1 ± 0.5 | 0.087 ± 0.026 | -11.4 ± 2.4 |
| Lipo-RSV-F127 1 | 143.5 | 0.13 | -6.62 |
| Lipo-RSV-F127 2 | 164.6 | 0.156 | -7.02 |
| Lipo-RSV-F127 3 | 134.0 | 0.17 | -11.1 |
| Mean | 145.3 ± 13.7 | 0.15 ± 0.25 | -8.25 ± 2.02 |

**[Table S6](#正文tableS6) The drug loading (DL%) and encapsulation efficiency (EE%) of Lipo-RSV**

|  | **Drug loading (DL%)** | **Encapsulation efficiency (EE%)** |
| --- | --- | --- |
| Lipo-RSV 1 | 82.21% | 3.98% |
| Lipo-RSV 2 | 85.54% | 4.08% |
| Lipo-RSV 3 | 76.17% | 3.60% |
| Mean | 81.30% ± 3.88% | 3.89% ± 0.25% |


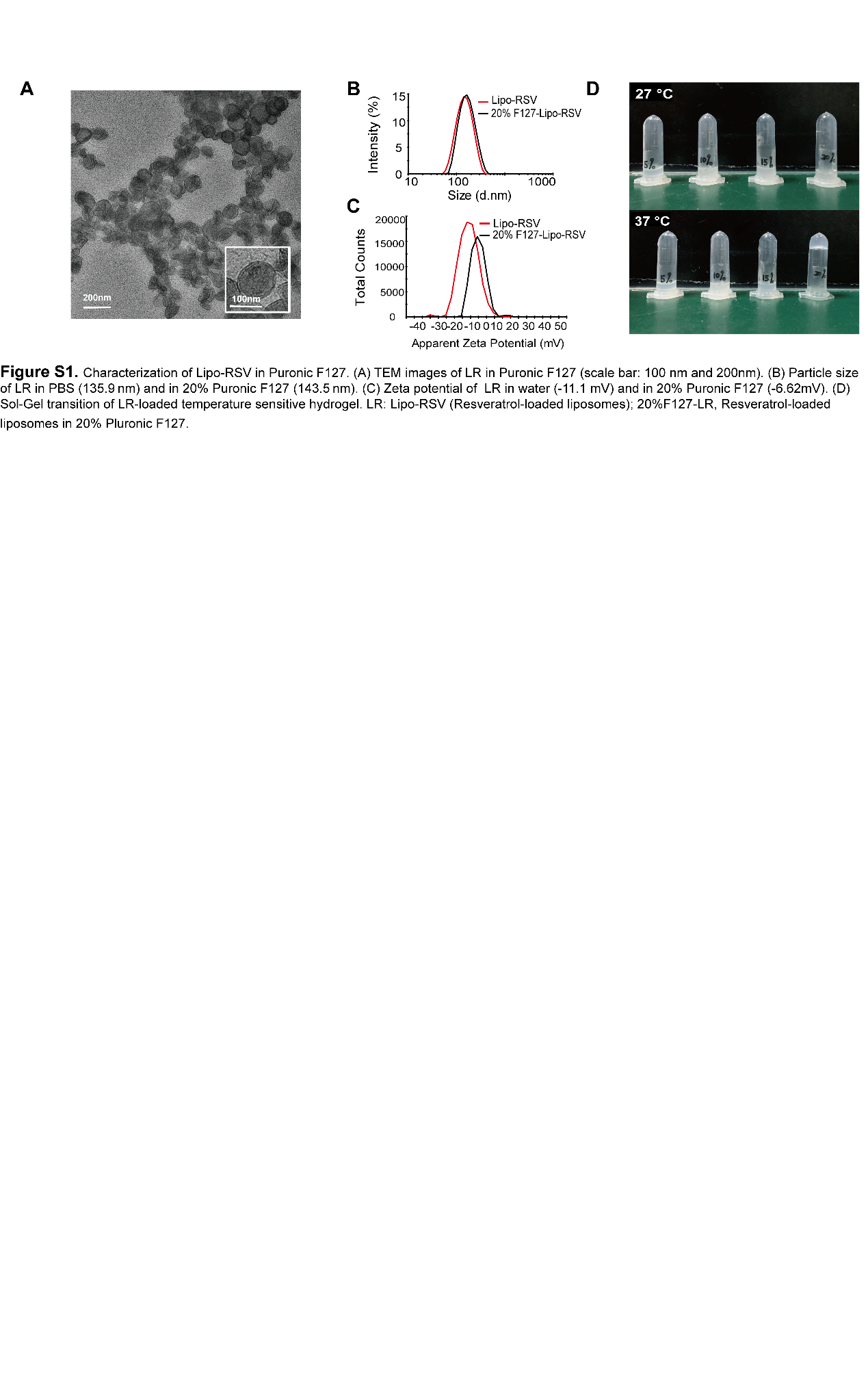
**[Figure S1](#正文figs1) Characterization of Lipo-RSV in Pluronic F127.** (A) TEM images of Lipo-RSV in Pluronic F127 (scale bar: 100 nm and 200nm). (B) The particle size of Lipo-RSV in PBS (135.9 nm) and in 20% Pluronic F127 (143.5 nm). (C) Zeta potential of Lipo-RSV in water (-11.1 mV) and in 20% Pluronic F127 (-6.62mV). (D) The sol-Gel transition of LipoRS

V-loaded temperature-sensitive hydrogel. Lipo-RSV: Resveratrol-loaded liposomes; 20% F127-Lipo-RSV, Resveratrol-loaded liposomes in 20% Pluronic F127.


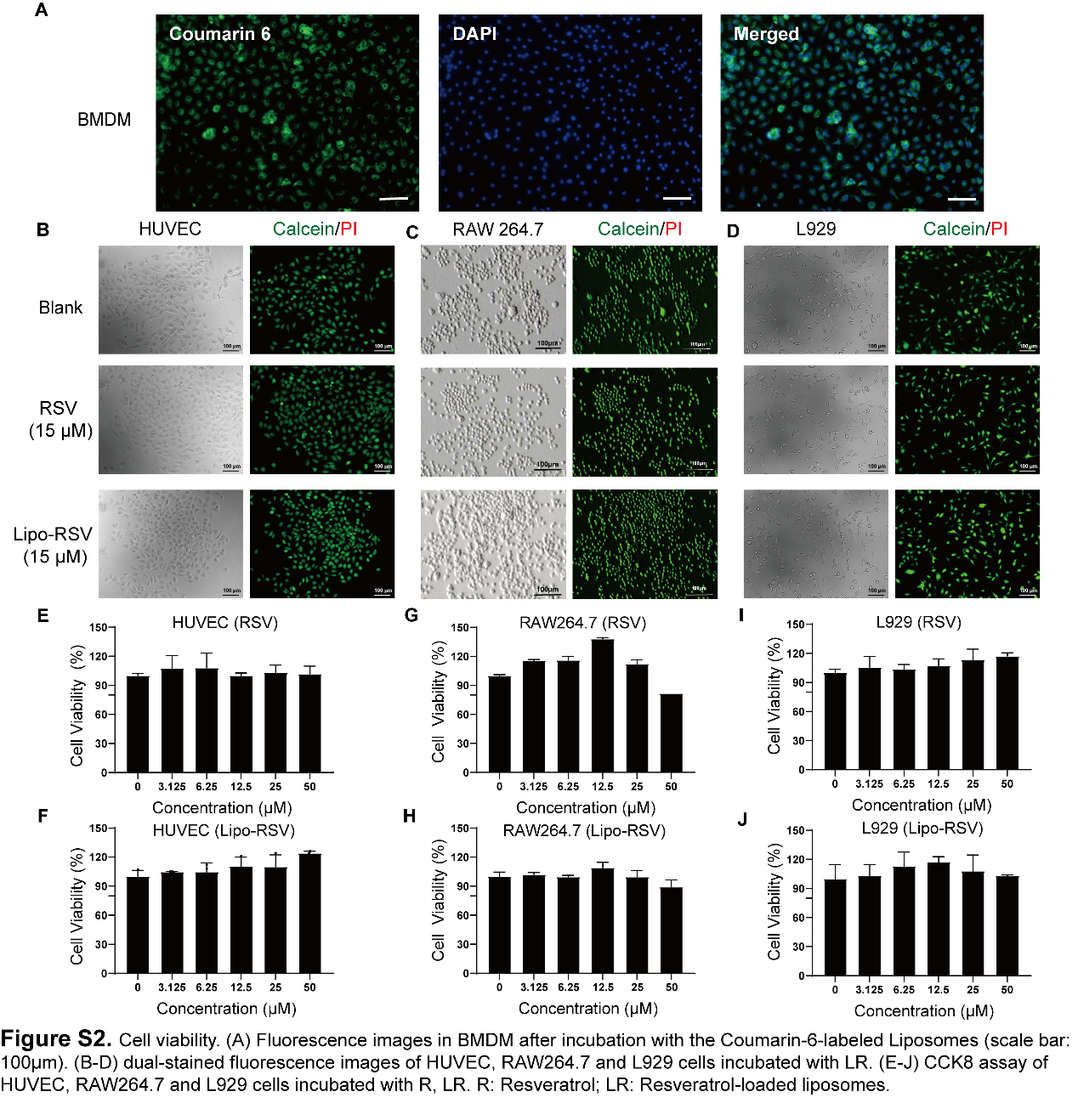


**[Figure S2](#正文figS2) Cell viability**. ([A](#正文figS2A)) Fluorescence images in BMDM after incubation with the Coumarin-6-labeled Liposomes (scale bar 100 μm). ([B-D](#正文figS2BD)) dual-stained fluorescence images of HUVEC, RAW264.7, and L929 cells incubated with Lipo-RSV. ([E-J](#正文figS2EJ)) CCK8 assay of HUVEC, RAW264.7, and L929 cells incubated with Lipo-RSV. RSV: Resveratrol; Lipo-RSV: Resveratrol-loaded liposomes.


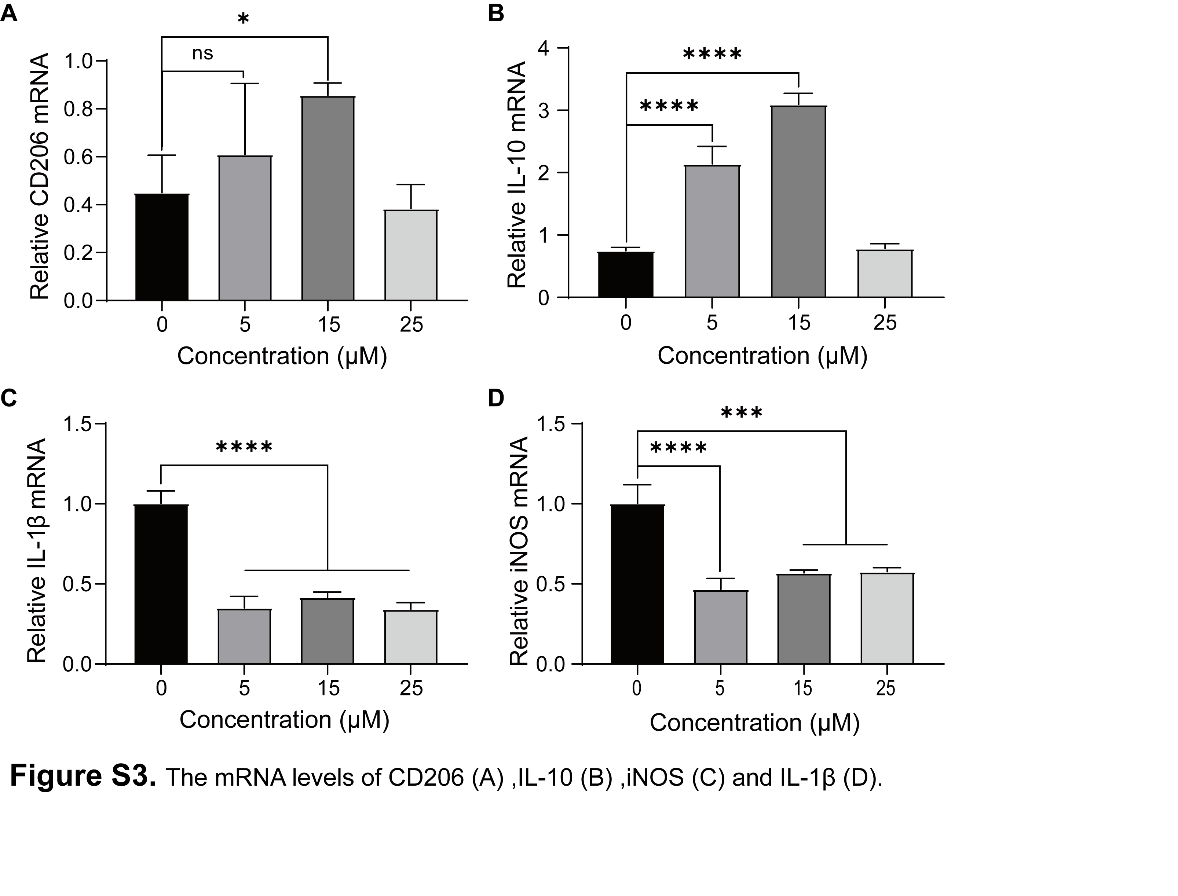


**[Figure S3](#正文figS3)** The mRNA levels of CD206 (A), IL-10 (B), iNOS (C), and IL-1β (D). Data are presented as mean ± SD (n = 3); ns, no significance, *P < 0.05, ***P < 0.001, ****P < 0.0001.


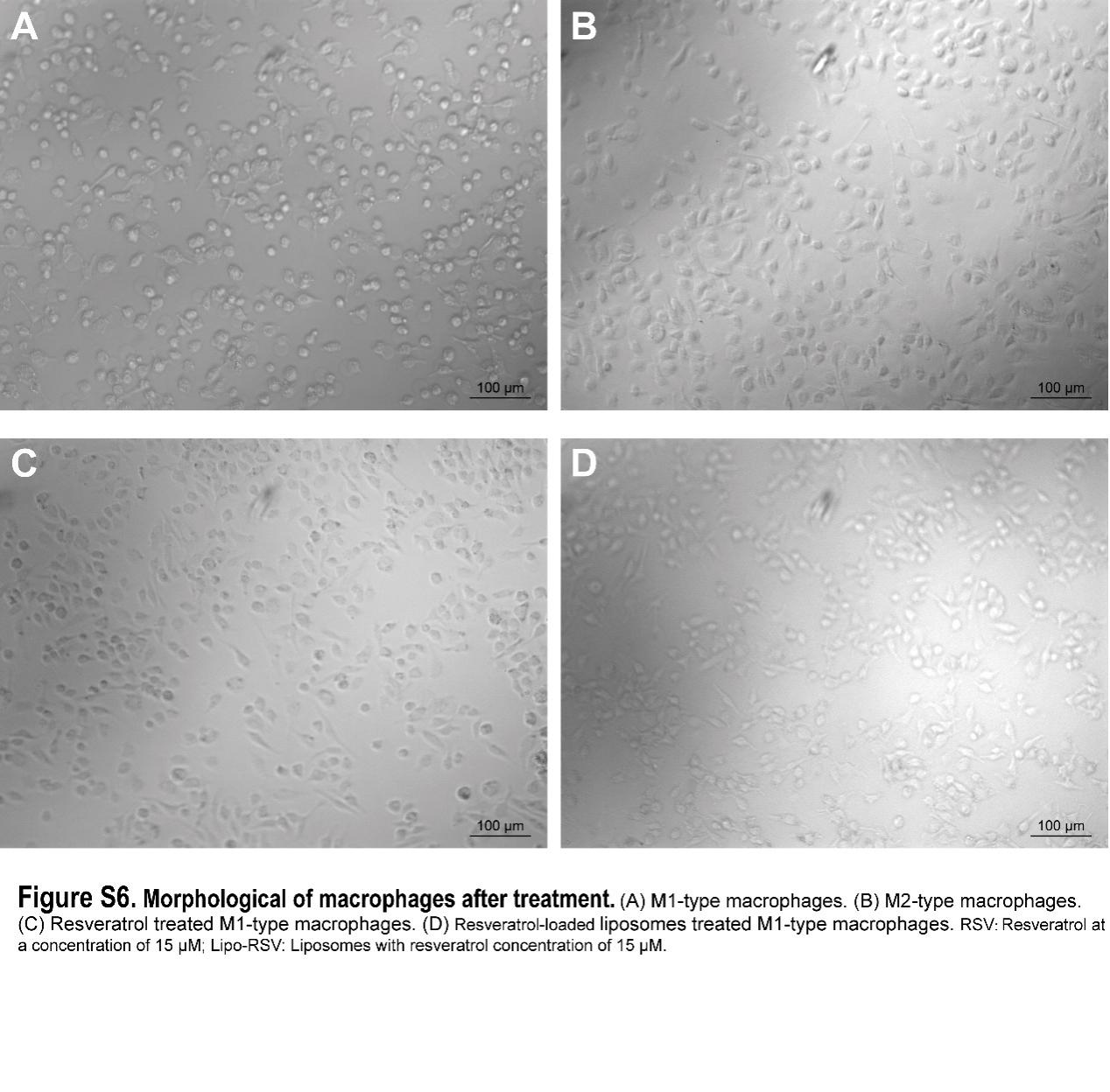


**[Figure S4](#正文figS5)** Cell morphology after treatment. (A) M1-type macrophages. (B) M2-type macrophages. (C) Resveratrol-treated M1-type macrophages. (D) Lipo-RSV-treated M1-type macrophages.


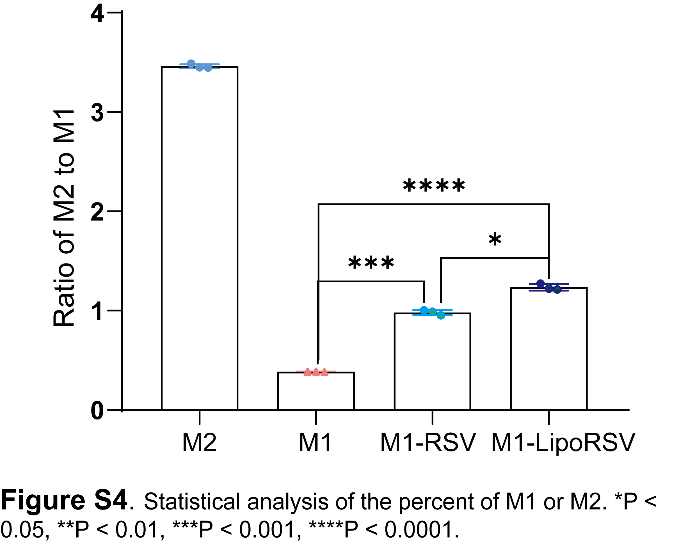


**[Figure S5](#正文figS4)** Statistical analysis of the percentage of M1 or M2. Data are presented as mean ± SD (n = 3); *P < 0.05, ***P < 0.001, ****P < 0.0001.


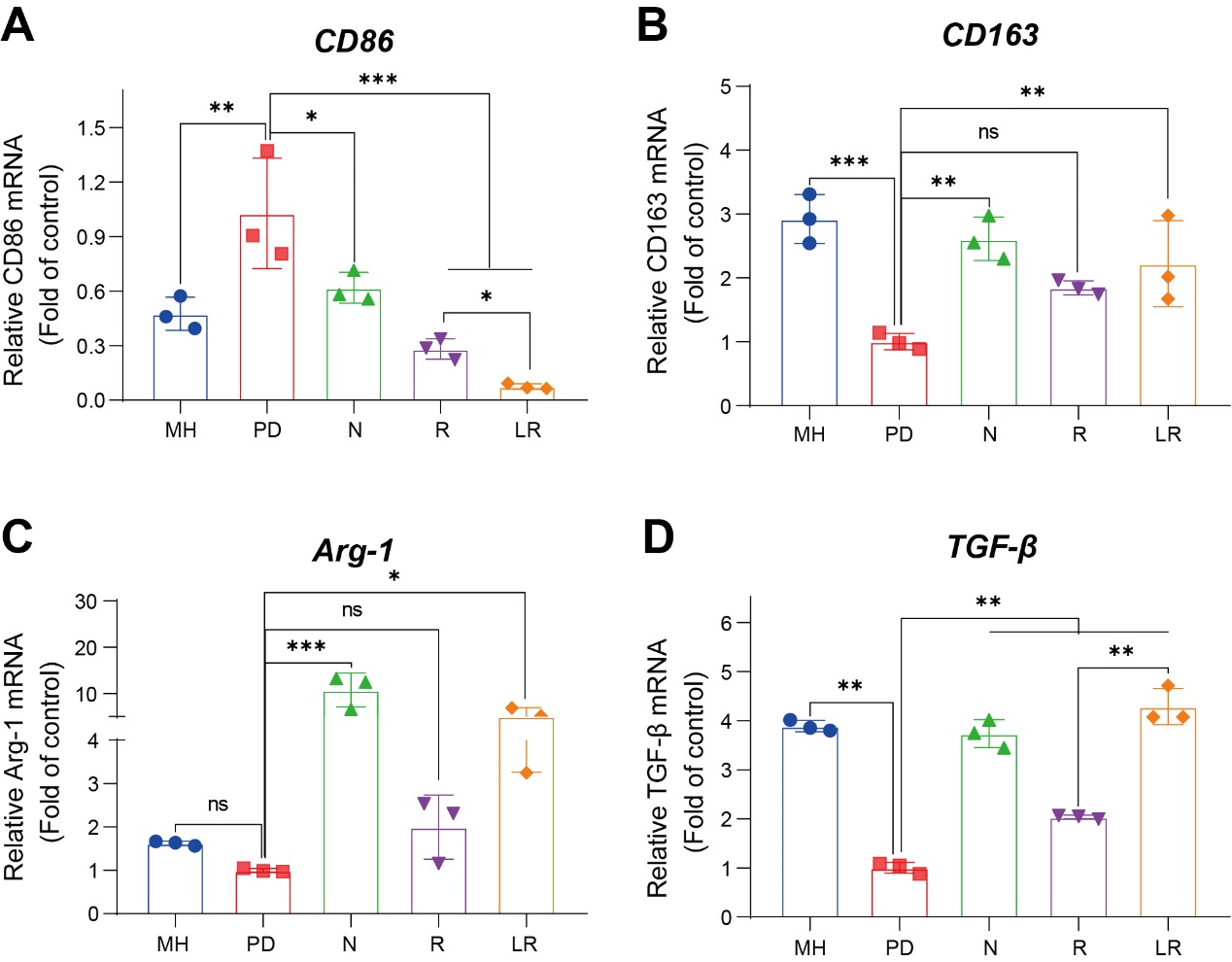


**[Figure S6](#正文FIGS6)** The mRNA level of M1 biomarkers (*CD86*) and M2 biomarkers (*CD163, Arg-1, TGF-β*) in gingiva were harvested from the mouse periodontitis model. Data are presented as mean ± SD (n = 3); ns, no significance, *P < 0.05, **P < 0.01, ***P < 0.001.


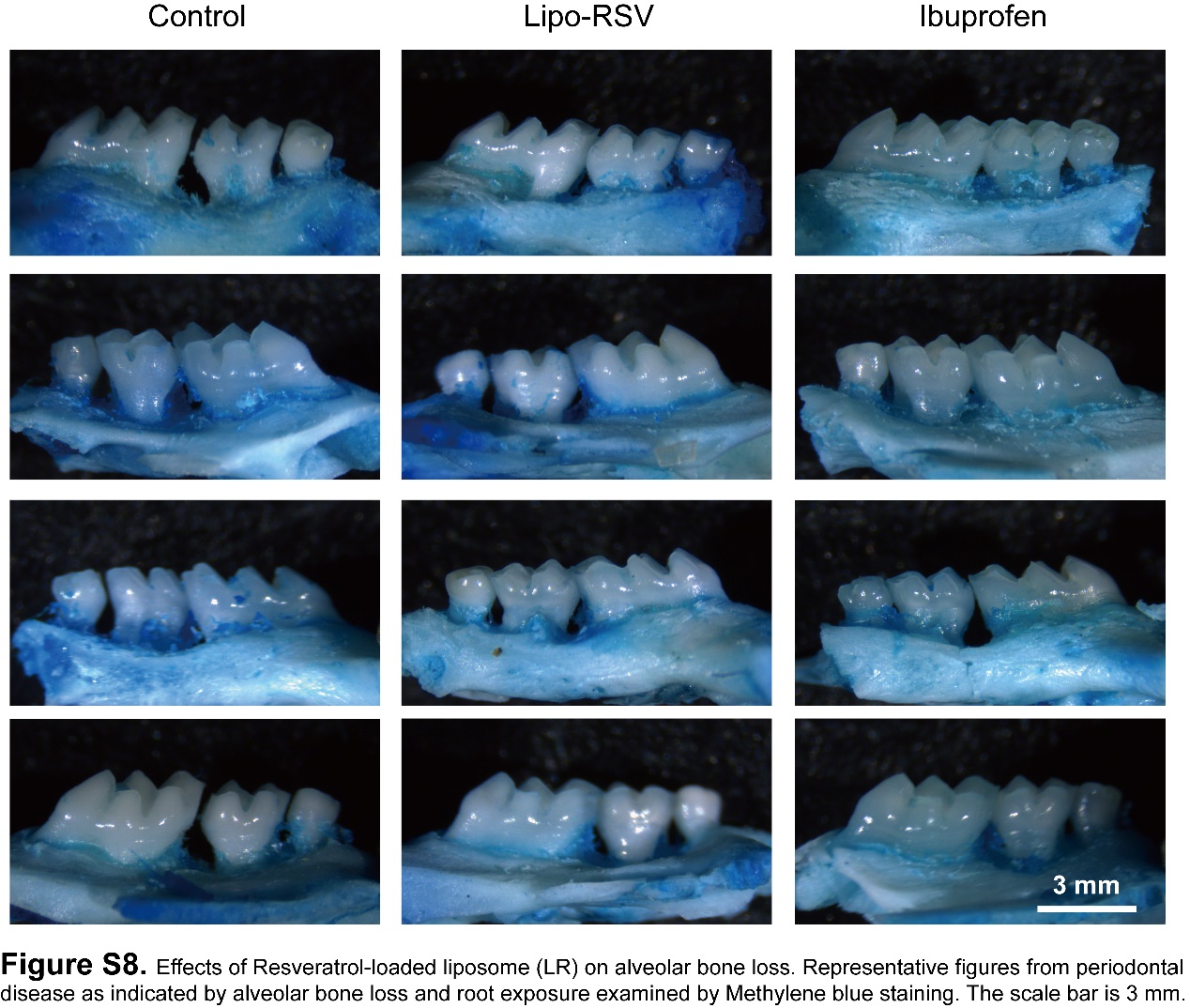


**[Figure S7](#正文FIGS7)** Effects of Lipo-RSV on alveolar bone loss. Representative figures from periodontal disease as indicated by alveolar bone loss and root exposure were examined by methylene blue staining.


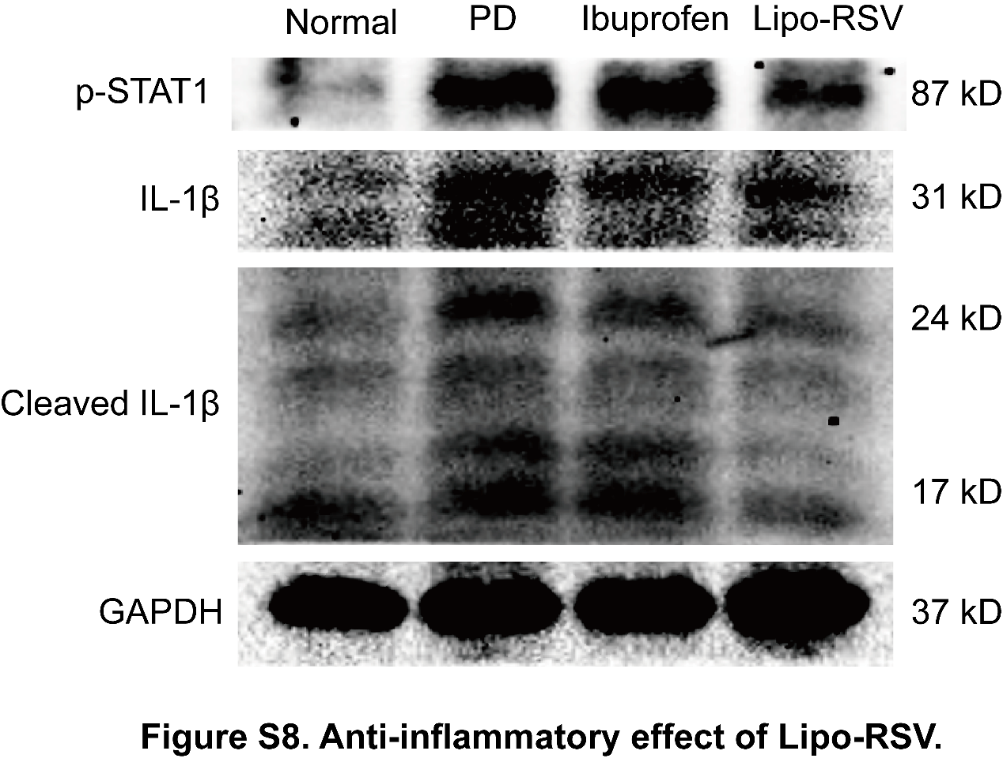


**[Figure S8](#正文figS8)** Anti-inflammatory effect of Lipo-RSV compared with ibuprofen.

**
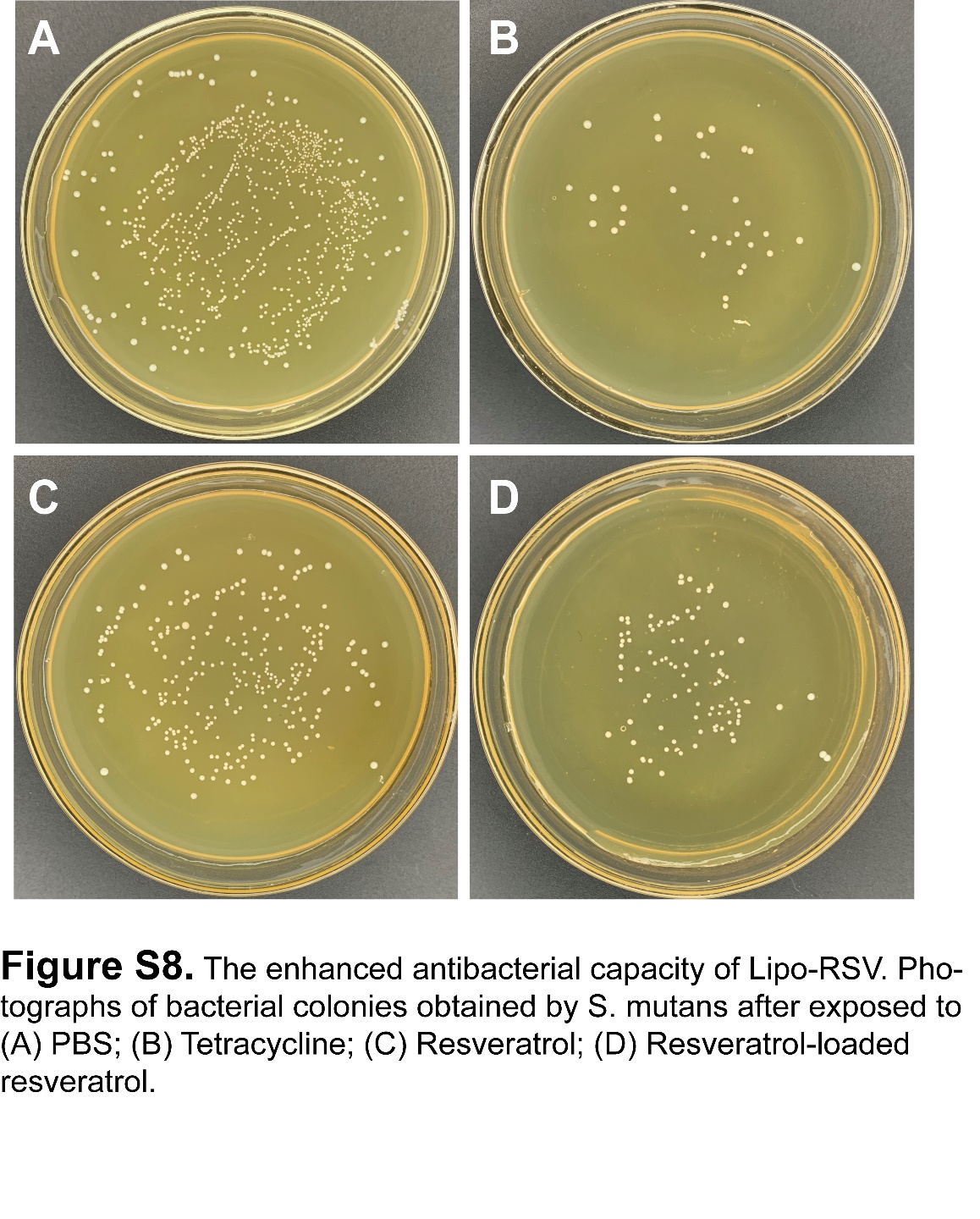
**

**[Figure S9](#正文figS9)** The enhanced antibacterial capacity of Lipo-RSV. Photographs of bacterial colonies obtained by S. mutans after exposure to (A) PBS; (B) Tetracycline; (C) RSV; (D) Lipo-RSV.


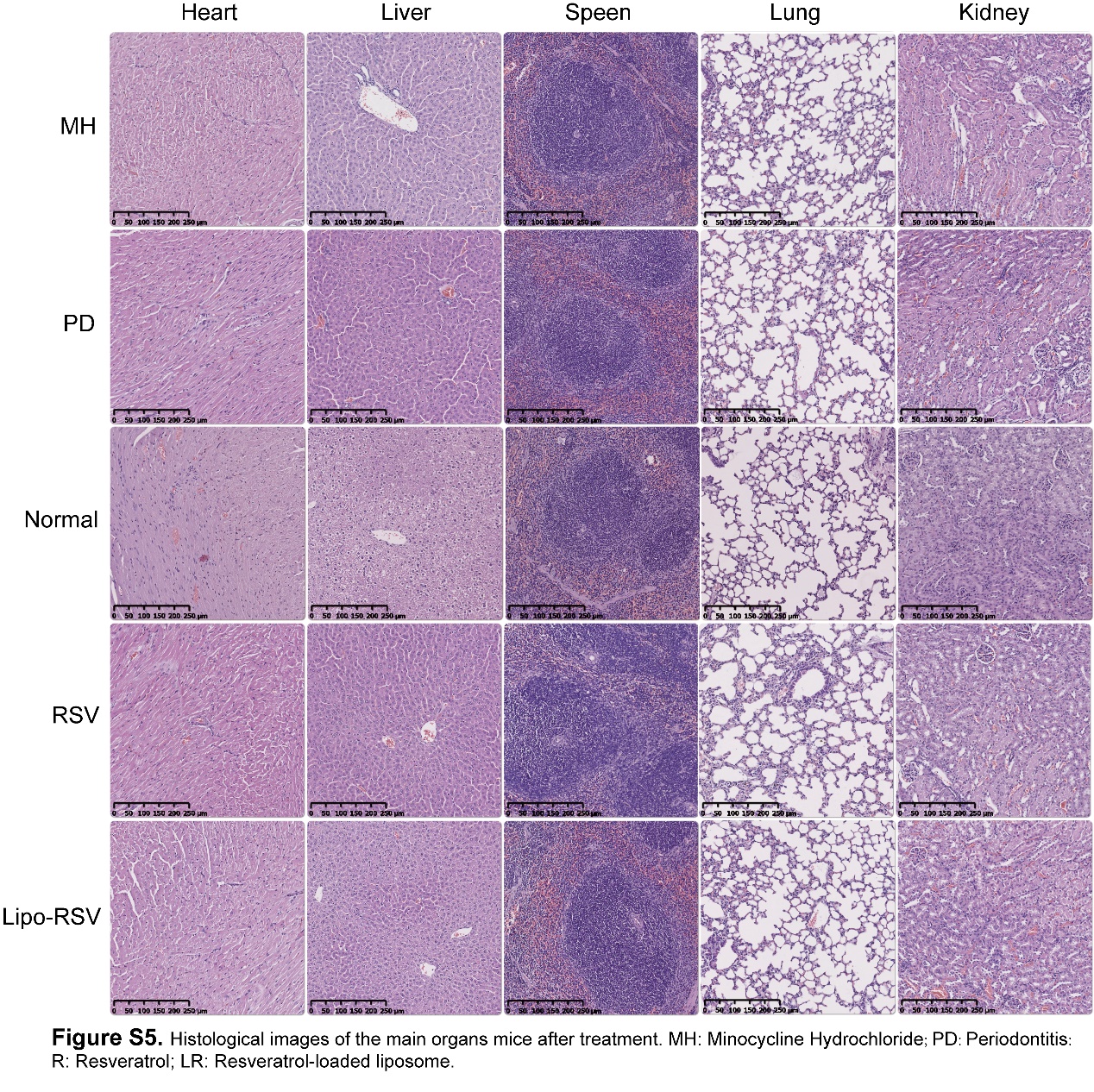


**[Figure S10](#正文FigS10)** Histological images of the main organs of mice after treatment. MH: Minocycline Hydrochloride; PD: Periodontitis: RSV: Resveratrol; Lipo-RSV: Resveratrol-loaded liposome.


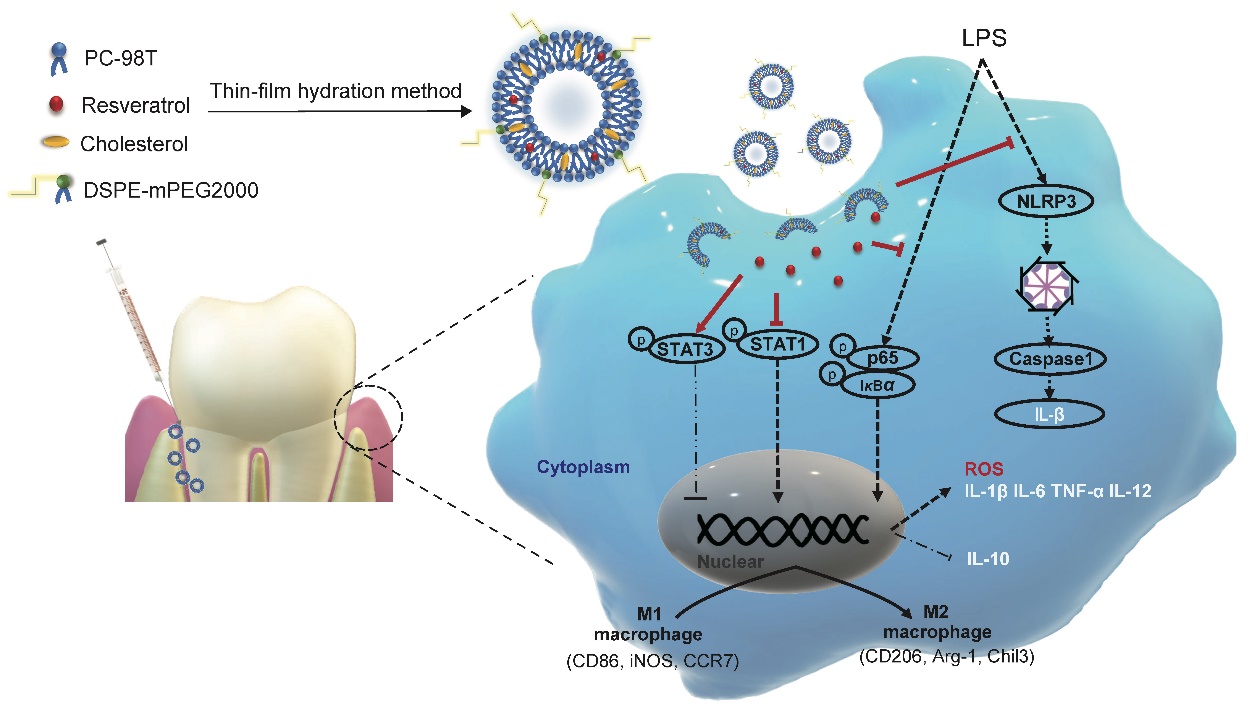

Supplement: Supplementary file 1 — Additional file 1. Additional Tables S1–S5 and Figures S1–S9. [file 12951_2021_1175_MOESM1_ESM.docx]
